# Ultrasound-guided femoral venous access decreases vascular complications in catheter ablation procedures

**DOI:** 10.1038/s41598-025-21481-w

**Published:** 2025-10-03

**Authors:** Friederike Pavel, S. C. R. Erlhöfer, J. Wörmann, S. Dittrich, C. Scheurlen, K. Filipovic, J.-H. Schipper, J.-H. van den Bruck, A. Sultan, J. Lüker, D. Steven

**Affiliations:** 1 Department of Electrophysiology, Heart Center of the University of Cologne, Cologne, Germany; 2 DRK-Hospital Berlin Westend, Berlin, Germany; 3 Asklepios-Hospital St. Georg, Hamburg, Germany

**Keywords:** Vascular complications, Pulmonary vein isolation, Ultrasound guided access, Atrial fibrillation ablation, Medical research, Outcomes research

## Abstract

Ultrasound (US) guidance is increasingly used in invasive cardiac electrophysiology (EP) procedures for femoral vascular access. In this study, we assessed the occurrence of vascular access-related complications in EP procedures which were performed with the routine use of anatomical landmark (LM) versus US-guided vascular access. A total of 1119 consecutive EP procedures in 1012 patients performed in a two-year period from September 10, 2020 to September 10, 2022 were included. The endpoint of the present study consisted of any vascular access-related complication, classified as hematoma, aneurysm, or AV-fistula. Different risk factors for increased bleeding risk were analyzed. During the evaluation period, 777 procedures were performed using LM-guiding and 342 procedures using US-guided access. Overall, 19 (1.7%) relevant vascular complications occurred including: 15 (1.3%) hematoma, 2 (0.18%) aneurysm and 2 (0.18%) AV-fistula. 17 (2.2%) complications occurred in the LM-guided group and 2 (0.6%) in the US-guided group. A significant reduction of femoral complications by 89% was observed with introduction of routine US-guided access. 3.8% in the LM-group vs. 0.4% in the US-group (OR 0.1, 95% CI 0.0135–0.8515, *p* = 0.034). Intraprocedural ACT and the HASBLED score [range 0–4; mean = 1.47; maximum = 4) were shown to be independent predictors for vascular complications (OR 2.826, 95% CI 1.631–4.895, *p* < 0.001). The use of US-guided vascular access significantly decreased the access-related complication rate in EP procedures. Higher procedural ACT and HASBLED score independently predicted a higher risk of vascular access complications.

## Introduction

The number of catheter interventions in cardiology, particularly in cardiac electrophysiology (EP), is rising^1^. Amongst the most common complications in EP procedures is vascular damage due to access related trauma. A meta-analysis of 89 RCTs found a procedure-related complication rate in catheter ablations for atrial fibrillation of up to 1.3%, representing 45% of all complications^2^. Complication rates vary depending on the type of the procedure: Catheter ablation aiming for pulmonary vein isolation (PVI) has the highest rate of vascular related complications (1.8% vs. non-PVI ablations 0.4%)^3^ and represents the highest volume of EP procedures.

The conventional method of landmark (LM) guided access by palpation of the femoral artery and puncturing medially to the femoral pulsation is still commonly used. However, accumulating evidence indicates that LM-guided access may be insufficient to locate and safely access the femoral vein reliably. Computer tomography (CT) studies showed that the femoral artery overlaps the femoral vein in two-thirds of examined cases^4^ with a high risk of accidental damage to the artery when performing a puncture without direct visualization.

Ultrasound guided access is an easily adoptable technique and detailed description of its application is readily available^5^. The use of ultrasound (US) to establish vascular access has the potential to lower the rate of major and minor vascular complications by approximately 60%^6^. Different risk factors for vascular complications after femoral access were examined with heterogenous results. The duration of applied compression after the procedure predicted the number of access related bleeding complications in one study^7^. Arterial access^8^ and a greater femoral vein depth^9^ (correlating with a higher BMI) were reported as risk factors associated with a higher complication rate. But especially regarding the role of BMI, antiplatelet therapy^7^ and gender^8,10^ disparate results were published.

There is a need to identify patients at risk and predictors of complications to further lower the risk of EP procedures, while measures to reduce procedural costs must also be considered as health care systems become increasingly strained and incremental expenses caused by complications can amount to €15,544.71 for certain complications^11^.

In earlier studies, including meta-analyses, US-guided access was associated with a 71% relative risk reduction of major vascular complications compared to standard technique^6,12^, reduced time to attain access, and improved outcomes for minor complications, accidental artery puncture and post-procedural pain^13^.

The present evaluation analyzed the potential advantages of US-guided access to decrease the number of vascular complications and identified further clinical risk factors of vascular access related complications in a clinical routine setting for EP procedures.

## Methods

### Study design

This analysis was designed as a retrospective, non-randomized, single-center trial aiming to investigate the potential advantages of US-guided access to decrease vascular complications, and to identify risk factors of vascular complications in patients undergoing catheter ablation of atrial fibrillation or other symptomatic supraventricular arrhythmia. We analyzed a total of 1119 consecutive EP procedures in 1012 patients performed from September 10, 2020 to September 10, 2022. An exclusive US-guided access regimen was implemented on September 10th 2021. The endpoint consisted of any vascular access-related complication classified as hematoma, aneurysm, or AV-fistula for two groups (conventional vs. US-guided access) within 4 weeks after the ablation procedure.

For further analysis risk factors including HASBLED Score, intraprocedural activated clotting time (ACT), use of platelet aggregation inhibitors or oral anticoagulants (OAC), age, diabetes mellitus and body mass index (BMI) were examined. The trial complied with the Declaration of Helsinki, the local ethics committee approved the protocol and all patients provided written informed consent for the procedure, general data acquisition, processing and analysis.

### Study population

We included patients over a period of two years who were scheduled for ablation of atrial fibrillation, and other types of symptomatic supraventricular tachycardias one year prior and one year after implementation of routine use of US-guided access at our center. Ablation procedures for premature ventricular contractions and ventricular tachycardia were not included in the analysis as the focus was on a consistent group of patients with a comparable peri- and intraprocedural anticoagulation regime.

### Vascular access

**Fig. 1:**
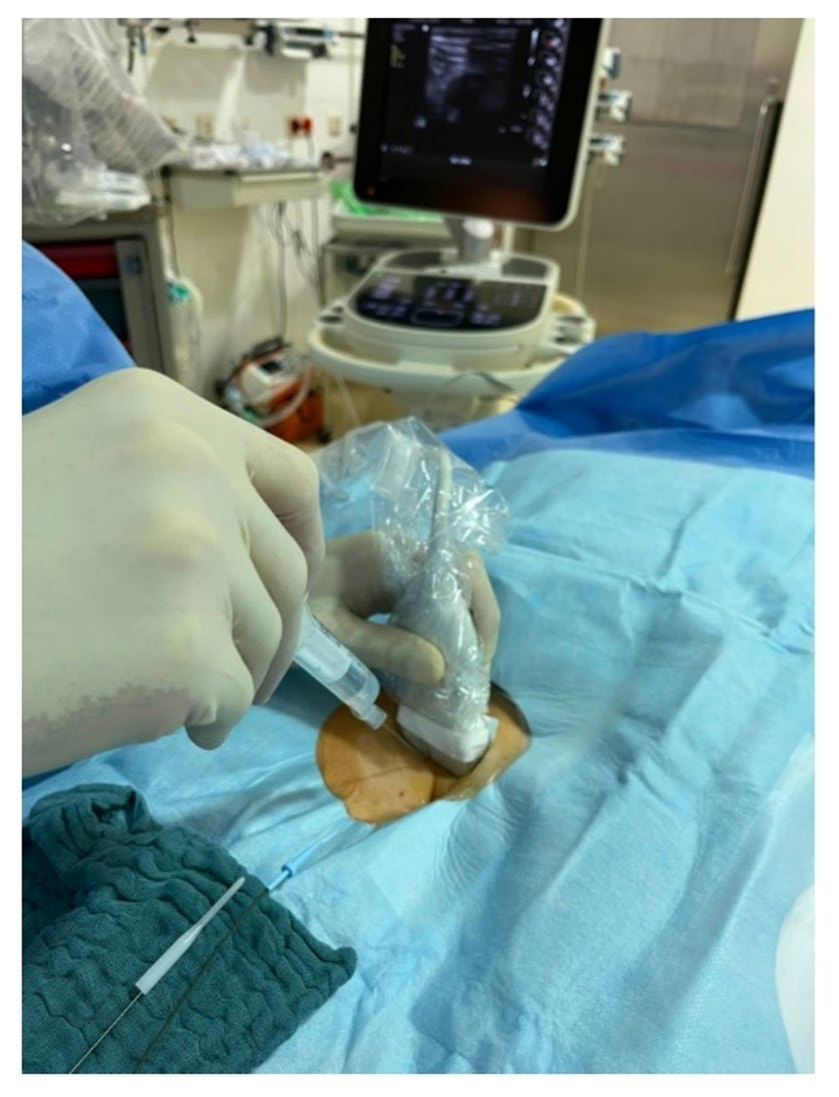
Venous access using ultrasound guidance via direct visualization. (Photo rights by J. Schipper MD).

**Fig. 2:**
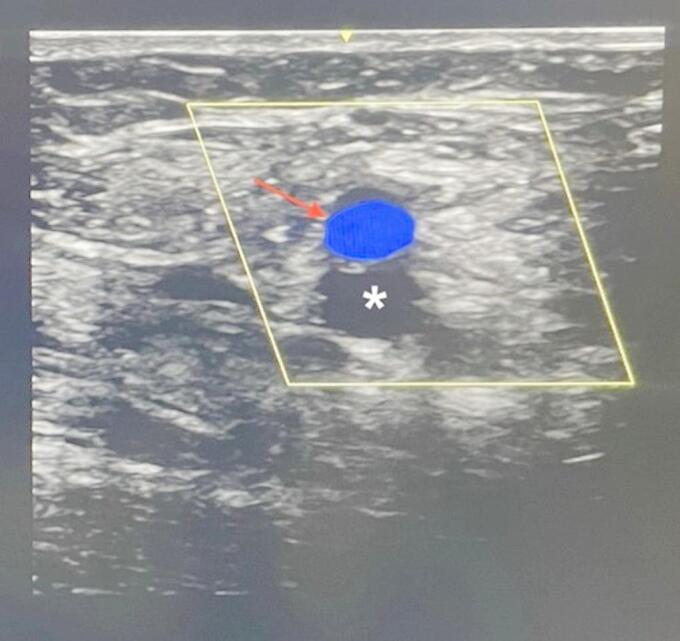
US-visualization of the right groin. Femoral artery (arrow) overlapping the femoral vein (asterisk). (Photo rights by J. Schipper MD).

All patients gave prior informed written consent and were prepared for the scheduled intervention by removal of body hair in the inguinal region and positioned in supine position. Fentanyl, propofol and midazolam were used to achieve deep sedation followed by the preparation of a sterile area by application of disinfectant solution (*Kodan*^®^, *Schuelke*,* Norderstedt*,* Germany*) and use of a fenestrated sterile drape (*Bisping*,* Aachen*,* Germany*). For the first group, LM guidance was performed by palpating the right femoral artery and puncturing medially to the palpated pulse. In the second group, access in the right femoral vein was established with direct visualization of the inguinal anatomy (Figs. 1 and 2). Identification of the vein using a linear ultrasound probe (*Philips Sparq*,* Hamburg*,* Germany* or *GE vivid E95*,* Boston*,* Massachusetts*,* USA*) with a sterile cover (*FlexaSoft*^®^, *Udo Heisig GmbH*,* Putzbrunn*,* Germany*) was secured by compression testing and color doppler signaling.

The number of inserted sheaths varied according to the procedure: four for atrial tachycardia or atrial flutter and three for PVI. For all procedures body-weight-adjusted unfractionated heparin (UFH) was administered with a calculated dose of 160 IE per kg in patients taking direct oral anticoagulants (DOAC). DOAC taken twice daily were withheld in the morning before the scheduled procedure and resumed in the evening of the same day. Oral anticoagulants taken once daily were also interrupted for one dose in the morning and resumed at half the dose in the evening. Patients taking vitamin K antagonists were instructed to maintain an INR between 2.0 and 2.5 on the day of the procedure. The UFH dose was determined and applied based on the INR (INR 1.0-1.3 > 100 U/kg, 1.3–1.9 75–100 U/kg, 1.9–2.3 75 U/kg, > 2.3 50–75 U/kg).

### Endpoint evaluation

After sheath removal, manual pressure at the puncture site for at least 10 min and figure of eight-sutures were routinely utilized followed by application of a compression bandage. A dedicated groin compression device (*FemoStop™*ฏ *Gold*,* Abbott*,* Lake County*,* Illinois*,* USA*) was applied in case of postprocedural bleeding after suturing at the discretion of the operator and applied for four hours following two to four hours of passive compression. Bed rest and immobilization were mandated for a minimum of 6 h for all procedures. Patients were then mobilized under supervision. Z-stich sutures were removed on the following day. During hospitalization, daily clinical visits including groin inspection and auscultation were performed in all patients followed by ultrasound of the groin in case of suspicious auscultation or clinical aspect. Patients were discharged after two nights post-procedure.

The endpoint of the study consisted of any vascular access-related complication, classified as hematoma, aneurysm, or AV-fistula. Relevant postprocedural bleeding and hematoma was defined in accordance with Bleeding Academic Research Consortium (BARC) criteria^14^ as greater than type 2 bleeding. AV-fistula and aneurysm were diagnosed using US and treated with manual compression followed by the application of a compression bandage.

### Statistical analysis

Data acquisition was conducted using an electronic data capture system (*RedCap Database*,* Nashville*,* Tennessee*,* USA*). The following statistical analysis was performed using IBM SPSS Version 28 (*SPSS*,* Chicago*,* Illinois*). Risk factors including HASBLED Score, intraprocedural ACT, OAC, use of platelet aggregation inhibitors, age, diabetes mellitus, BMI and sex were analyzed using binary logistic regression analysis and reported as Odds Ratio (OR) with a 95% confidence interval. Linearity was tested assessed using the Box-Tidwell procedure and Bonferroni-correction was applied to all terms in the model. All variables in the model were shown to follow a linear relationship. Correlations between predictor variables were low (*r* < 0.70), indicating that multicollinearity was not a confounding factor in the analysis.

The binomial logistic regression model was statistically significant (χ²(9) = 22.152, *p* = 0.008) and presented with a good model fit (χ²(8) = 8.434, *p* > 0.05) in Hosmer-Lemeshow-Test. Correlation analysis was conducted using nonparametric testing of Spearman’s Rho. A p-value less than 0.05 was considered significant. Fishers exact test was used as the observed cases in the 2 × 2 crosstabulation were less than five and tested as one-tailed hypothesis. Association analysis using Fisher‘s exact test revealed a significant association between US-access and vascular complications (*p* = 0.040) with negative effect size presented as ϕ= -0,057 that showed a lower complication rate in patients after US-guided groin access.

## Results

During the study period, 777 procedures were performed using anatomical LM-guided and 342 procedures using US-guided access. Mean age at time of procedure was 67.2 years (range 20–93) with a mean body mass index of 27.1 kg/m^2^ (range 17–52). Baseline characteristics of the enrolled patient cohort are presented in Table 1.

**Table 1:** Descriptive baseline characteristics.

	*N*	%	Mean	Range
Sex (male)	685	61.2		
Sex (female)	434	38.8		
OAC	983	87.8		
Antiplatelet therapy	41	3.7		
Diabetes	168	15.0		
Renal insufficiency	34	3.0		
Hepatic insufficiency	10	0.9		
History of bleeding	18	1.6		
Labile INR	6	0.5		
Hypertension > 160 mmHg	75	6.7		
Age (years)			67.2	20–93
BMI (kg/m²)			27.1	17–52
ACT (sec)			256	174–320
HASBLED Score			1.47	0–4

ACT = activated clotting time. HASBLED = Hypertension, Abnormal renal/liver function, Stroke, Bleeding history or predisposition, Labile INR, Elderly, Drugs/alcohol concomitantly.

**Fig. 3:**
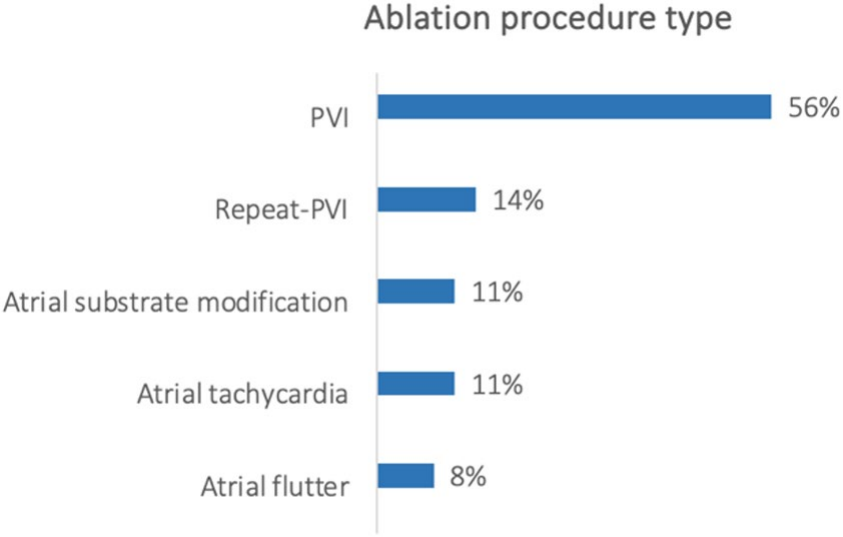
Ablation procedure types. Ablation procedures percentage of total ablation number devided by procedure type.

**Fig. 4:**
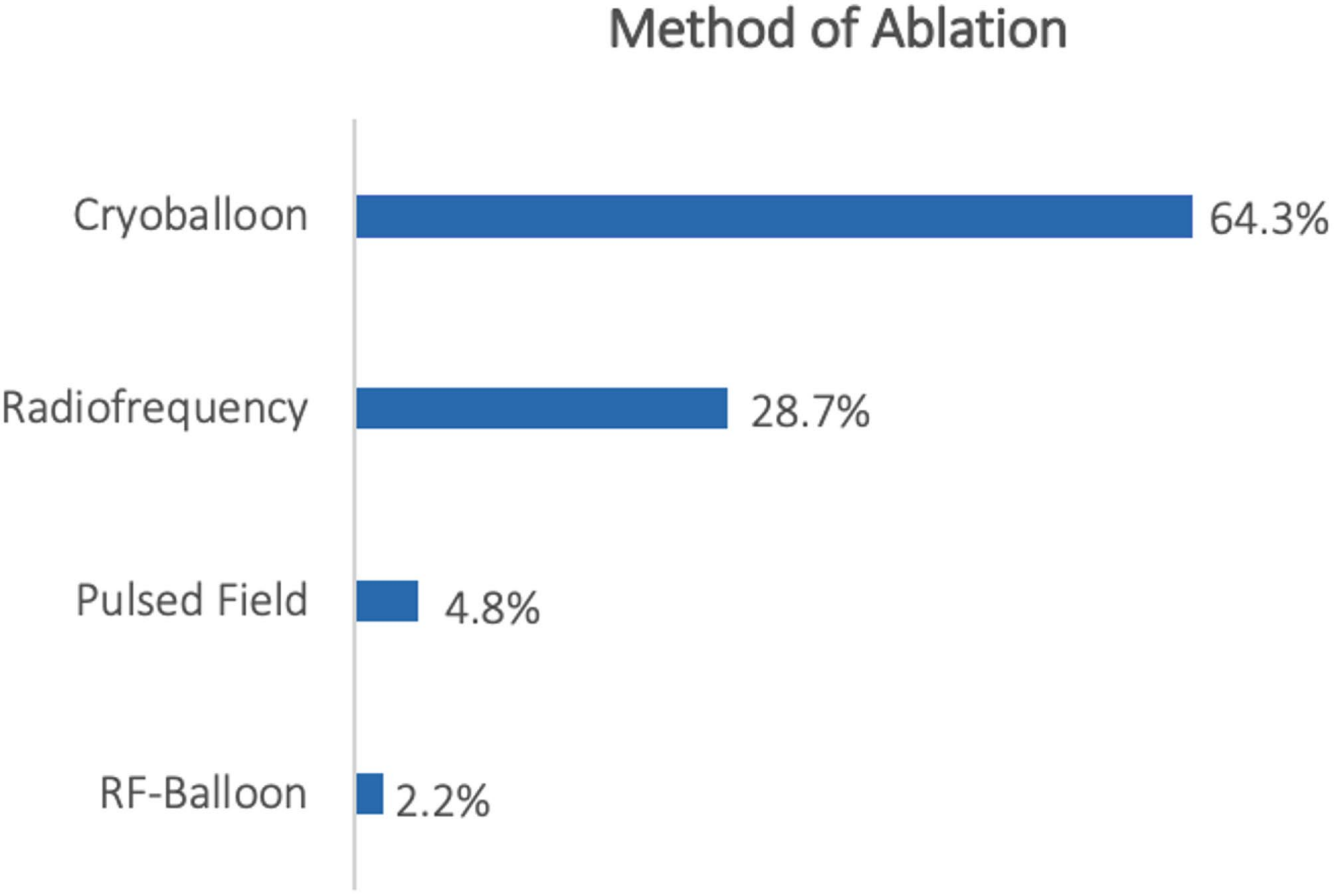
Method of ablation. Ablation procedures divided by method of ablation technique.

The examined procedures (Fig. 3) were performed for PVI (56%), Repeat-PVI (14%), atrial substrate modification in AF (11%), atrial tachycardia (11%) and atrial flutter (8%). In most cases a cryo-balloon technique was used (64.3%), followed by radiofrequency ablation (RF, 28.7%), pulsed field ablation (4.8%), and RF-balloon (2.2%) as shown in Fig. 4.

**Fig. 5:**
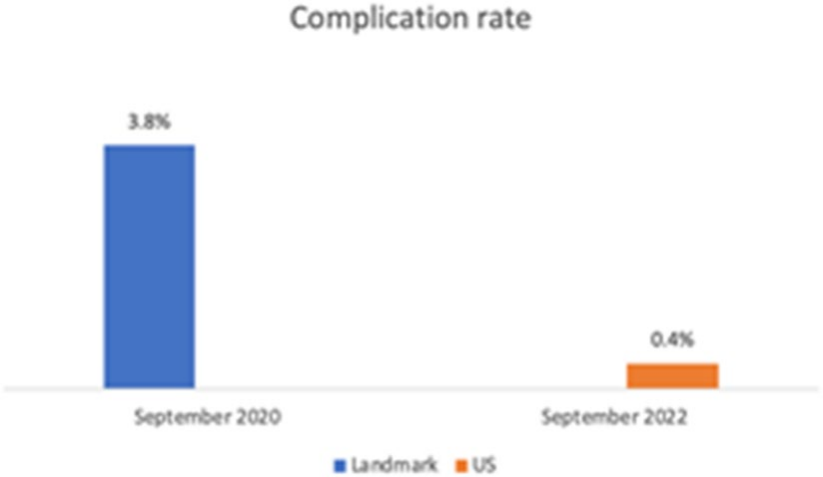
Complication rate. Vascular complication rate before and after implementation of US-guided access.

Overall, 19 (1.7%) relevant vascular complications meeting the above-mentioned criteria occurred, including: 15 (1.3%) hematoma, 2 (0.18%) aneurysm, and 2 (0.18%) AV-fistula. 17 (2.2%) complications occurred in the control group using LM-guided access and 2 (0.6%) in the US-access group. The results indicate a significant association between US access and decreased vascular complications (*p* = 0.040 ϕ= -0.057) with a reduction of the observed complication rate by 89%: 3.8% complication rate in the LM-guided group vs. 0.4% in the US-guided group (OR 0.1; 95% CI 0.0135–0.8515, *p* = 0.034) as depicted in Fig. 5.

Moreover, complications in the US-guided group were less severe: Both cases presented with hematoma, no cases of AV-fistula or aneurysm were observed. One case of arterial pseudo-aneurysm in the landmark group required surgical intervention.

Regression analysis demonstrated that only HASBLED Score count (range 0–4; mean = 1.47; maximum = 4, OR 2.826, 95% CI 1.631–4.895, *p* < 0.001) and the first intraprocedural ACT (activated coagulation time, range 174–320 s, mean = 256 s, OR 0.985, 95% CI 0.972–0.998, *p* = 0.019) were independent predictors of the 19 vascular complications.

Regarding ACT values, no significant difference in heparin dosage (13141.3 Units vs. 12277.7 Units; *p* = 0.120) or dosage per kg bodyweight (mean 156.9 U/kg vs. 148.2 U/kg; *p* = 0.263) was found in patients who suffered a complication.

Maximum ACT was 320 s in patients with a diagnosed vascular complication (range 174–320 s; mean 256 s) and the maximum HASBLED Score count was 4 (range 0–4; mean 1.47).

Oral anticoagulation, use of platelet aggregation inhibitors, BMI, prediagnosed diabetes mellitus, age at time of procedure and sex did not predict the number of complications (Table 2).

**Table 2:** Predictor analysis for vascular complication.

	OR	95% CI	*p*-value
Sex (male)	0.549	0.211–1.430	0.220
OAC	0.980	0.158–6.075	0.982
Antiplatelet therapy	0.749	0.069–8.195	0.813
Diabetes	4.085	0.467–35.74	0.203
Age (years)	0.956	0.910–1.004	0.074
BMI (kg/m^2^)	1.051	0.954–1.159	0.316
ACT (sec)	0.985	0.972–0.998	**0.019**
HASBLED	2.810	1.634–4.832	**< 0.001**

BMI, body mass index; CI, confidence interval; OAC oral anticoagulant; OR, odds ratio; HASBLED, hypertension, Abnormal renal/liver function, Stroke, bleeding history, labile INR, Elderly, Drugs.

Significant values are in bold.

## Discussion

The results of the present study confirm that adverse vascular events are infrequent in EP procedures. Still, the implementation of routine use of US-guided access significantly reduced the occurrence of vascular complications. In addition, we found that a higher ACT and higher HASBLED Score count were independent predictors of vascular complications in patients undergoing EP ablation procedures.

### US-guided access

Association analysis showed a lower complication rate after US-guided groin access as comparable to other studies with a similar sample size^13^. The utilization of ultrasound provides an easy to use and cost-effective method that does not prolong the procedure^13^. Previous studies have calculated the incremental expenses caused by complications to be up to €15,544.71 per patient^11^. Adopting US-guided access may decrease health care costs through its potential to reduce the number of vascular complications.

### Outcomes

**Fig. 6:**
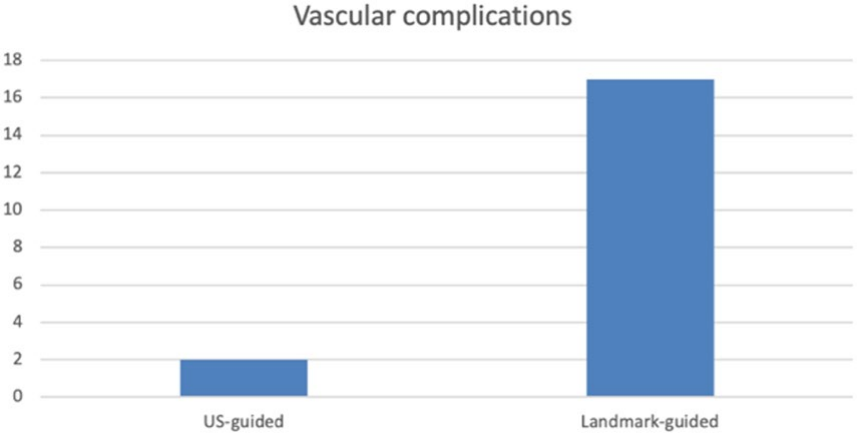
Number of Vascular complications. Number of vascular complications grouped by selected access.

We observed 17 (2.2%) complications in the LM access group and 2 (0.6%) in the US-guided access group (Fig. 6) over the course of the study. The most significant decrease was observed at the beginning of the study with a complication rate of 3.8% before the implementation of US-guided access compared to 0.4% after the transition to a US-only regime by the end of the study (OR 0.1, *p* = 0.034) corresponding to a reduction of 89%.

The variations in group sizes stem from the sequential nature of the real-world study design and the gradual transition from traditional landmark-guided to ultrasound-guided puncture methods following the implementation of the Standard Operating Procedure (SOP). Additionally, the occurrence of a significant work strike by the nursing staff led to a reduction in interventions during the specified timeframe after the release of the SOP, therefore delaying the implementation of a US-only approach resulting in an uneven cohort size. Furthermore, alongside correlation analysis of the entire cohort, we conducted comparisons between the initial and final phases of the observation period to account for any potential learning effects among the investigators and the unequal cohort size.

Observed complication rates in the US-group are in line with findings of other study groups^6^ and indicate a reassuringly low risk of harm to the patient during ablation procedures. Moreover, less severe complications were observed in the US-guided access group: both cases presented with hematoma which did not require therapeutic intervention, while in the control group two cases of AV-fistula and two aneurysm were observed, requiring surgical intervention in one. These results favor the use of US guidance to improve patient safety.

While the Yamagata et al. conducted the first randomized trial comparing an US-guided vs. a conventional access approach found no significant difference in major complications due to a lower than expected number of access-related complications^13^, a broader body of evidence has shown the benefits of the US-approach^15^. The current ESC-guideline for the treatment of atrial fibrillation therefore recommends the use of US-guided vascular access for all interventional ablation procedures^16^.

#### Anticoagulation and intraprocedural ACT

Encouragingly, the use of oral anticoagulation or antiplatelet therapy were no predictors of vascular complications, but our protocol dictated to pause the administration of oral anticoagulants prior to ablation must be taken into account when interpreting these results.

We found a negative correlation between vascular complications and the first measured ACT during ablation, though no significant difference in the absolute or bodyweight adjusted heparin dosage was present. We found no difference in ACT levels between different types of DOAC, with apixaban being the most commonly used DOAC. Different levels of confounding factors in the coagulation cascade might be at play in addition to varying levels of interacting plasma-binding proteins.

#### HASBLED score as independent predictor of vascular complications

We identified the HASBLED score as a predictor of vascular complications in patients undergoing ablation. The HASBLED score count is a readily available tool for risk factor assessment in all patients presenting with AF. Previous studies have found an effect of renal insufficiency as predictor of complications^3,17^ after AF ablation, which may at least partially explain the impact of the HASBLED score count. Nonetheless, only 3% of patients in our cohort suffered from a severe renal insufficiency. The increased risk for bleeding complications indicated by a high HASBLED score can also be applied specifically to the individual risk for vascular groin complications in patients receiving ablation for atrial fibrillation.

### Limitations

This was a retrospective, non-randomized, single-center study designed to identify clinical predictors of vascular complications in a group of consecutive patients undergoing EP procedures. Therefore, our findings cannot be applied to the general population. Despite the study’s retrospective nature, we sought to reduce selection bias by gathering information on consecutive patients who received EP procedures in our center. Nevertheless, selection bias regarding BMI is possible since our protocol comprises mandatory weight loss for patients with a BMI ≥35 with the indication for AF ablation and in the absence of arrhythmia induced cardiomyopathy or very severe symptoms (EHRA stage IV) before scheduling AF ablation. As a result, severely obese patients are underrepresented in this study.

Also, our study did not account for a potential learning curve of the operator. We presumed this to be negligible due to experienced expert level operators who had prior experience using ultrasound. Therefore, no specific training was mandated. Previous studies took this fact into account but did not find a significant difference in the performance of expert and less experienced electrophysiologists^13^.

Lastly, the respective cohort sizes were not ideally equal between both groups (LM vs. US-guided) as the presence of a notable strike by the nursing staff resulted in decreased interventions during the study, consequently yielding a diminished ultrasound group. Nevertheless, post-hoc power analysis verified an acceptable sample size (87.4% power at an alpha niveau of 5%).

## Conclusion

The use of ultrasound-guidance for vascular access significantly further reduced the rate of groin complications in this retrospective analysis of EP procedures. Therefore, adopting the US-guided vascular access method into routine practice should be considered.

To minimize the risk of vascular complications, it is essential to screen patients who present with a high HASBLED Score count. Tailored heparin dosage and conducting frequent ACT checks during ablation is advisable.

## Data Availability

The datasets generated during and/or analysed during the current study are available from the corresponding author on reasonable request.
